# Real-Time Classification of Ochratoxin a Contamination in Grapes Using AI-Enhanced IoT

**DOI:** 10.3390/s25030784

**Published:** 2025-01-28

**Authors:** Mohamed Riad Sebti, Zohra Dakhia, Sonia Carabetta, Rosa Di Sanzo, Mariateresa Russo, Massimo Merenda

**Affiliations:** 1 Department of Information Engineering, Infrastructure and Sustainable Energy (DIIES), University Mediterranea of Reggio Calabria, 89124 Reggio Calabria, Italy; riad.sebti@unirc.it (M.R.S.); zohra.dakhia@unirc.it (Z.D.); 2 Department of Agraria, University Mediterranea of Reggio Calabria, 89124 Reggio Calabria, Italy; sonia.carabetta@unirc.it (S.C.); rosa.disanzo@unirc.it (R.D.S.); mariateresa.russo@unirc.it (M.R.); 3 HWA srl, Spin-Off Mediterranea University of Reggio Calabria, Via R. Campi II tr. 135, 89126 Reggio Calabria, Italy

**Keywords:** ochratoxin, edge computing, Internet of Things, agrifood

## Abstract

Ochratoxin A (OTA) contamination presents significant risks in viticulture, affecting the safety and quality of wine and grape-derived products. This study introduces a groundbreaking method for early detection and management of OTA, leveraging environmental data such as temperature and humidity. A function derived from chemical analysis was developed to estimate OTA concentrations and used to label a synthetic dataset, establishing safe thresholds. Two AI models were trained: one for the detecting of OTA presence and the other for classifying the concentration range. These models were deployed on a M5Stick C+, a microcontroller designed for real-time data processing. The inference process is optimized for rapid response, requiring minimal time to deliver results. Additionally, the low power consumption of the M5Stick C+ ensures that the device can operate throughout the harvest period on a single charge. The system is able to transmit inference data via MQTT for real-time analysis. This comprehensive approach offers a scalable, cost-effective, on-site solution that is autonomous, eliminating the need for domain experts and extensive resources. The robustness of the system was demonstrated through its consistent performance across multiple test sets, providing an effective enhancement to food safety in grape and wine production. The study also details the system architecture, describes the function used for data labeling, outlines the training and deployment processes of the models, and finally, assesses the testing of the overall system.

## 1. Introduction

Mycotoxins are poisonous substances that are naturally created by some molds (fungi) and are present in a variety of foods, including cereals, nuts, dried fruits, and spices. Mycotoxin-producing molds can grow on a wide variety of food products, and this unwanted event can happen before and after the harvesting of food products, during storage, and more frequently in warm, humid environments [1,2]. Mycotoxins can enter the circulation and lymphatic system by ingestion, skin contact, inhalation, and other means, and they can have both acute and long-term health impacts [3]. They impair macrophage systems, prevent the lung from clearing particles, impede protein synthesis, and make the body more susceptible to bacterial endotoxins [4]. Immunoaffinity columns can be used to test for mycotoxicosis. The kind of mycotoxin, the amount and duration of exposure, the exposed person’s age, health, sex, and other factors all affect the mycotoxicosis symptoms [5]. Ochratoxin A (OTA) and Fusarium toxins, like deoxynivalenol, are the most frequent mycotoxins that are harmful to the health of people or animals. In addition to the exposure time, there are two key factors that affect the growth of these mycotoxins: temperature and humidity [6]. According to this fact, the continuous and smart monitoring of the conditions that facilitate the growth of these compounds can play a fundamental role in preventing contaminations and assuring health safety [7]. To this end, Internet of Thing (IoT) devices could play a fundamental role due to the reduced size, the low-power consumption, the availability of low-cost sensors, and the connectivity capabilities. This makes IoT a preferable candidate to design on top a proper system to support agrifood improvements related to the quality and safety of human-intended food products [8]. In wines, OTA is the most studied mycotoxin, and the European Commission (by regulation 1881/2006) [9] established, as the maximum tolerable level in grape juice, must, and wines destined for human consumption, a concentration of 2 µg kg^−1^. Focusing on the wine-production process, considering the stringent regulation, in this work, we propose a suitable IoT system that is able to estimate the presence and to provide a level classification of the growth probability of specific mycotoxins, using only low-cost sensors and exploiting the use of edge computing to identify environmental-related behavior of the monitored food environment. Mycotoxin contamination issues can be effectively addressed by integrating Artificial Intelligence (AI) and IoT [10] solutions in the grape sector. Nowadays, AI is becoming a key player in the digital revolution of the grape sector, offering insightful data on measurable characteristics and other vineyard circumstances. Winegrowers can now make data-driven decisions to increase grape yield thanks to AI, sensors, and visual data [11]. Furthermore, precision agriculture is made possible by the combination of AI with cultivation and vineyard management, giving vineyard managers unmatched control over the plants’ growth environment [12]. Vineyards can monitor soil health, climate conditions, and plant health in real-time by integrating AI with technology like drones and IoT devices [13,14,15,16]. This allows for the cultivation of superior grapes [17]. In order to guarantee ideal growing conditions, the integration of IoT sensors in vineyards also makes it possible to monitor environmental parameters like temperature, light intensity, and humidity in real-time. Grape producers can increase grape quality by making data-driven decisions with the help of this real-time information [18,19]. Furthermore, Chee and al. [20] present a solution that highlights the importance of IoT in tackling mycotoxin concerns in the grape business, which is crucial in the early detection of fungal development and OTA contamination.

We previously proposed in [21], with a specific focus on Machine Learning (ML) classification algorithm development, a method to classify the probability of OTA contamination. In that work, we trained a model with the usage of a synthetic generated dataset exploiting a custom labeling algorithm.

In this context, our work presents an innovative solution by integrating IoT technology with AI to address the issue of OTA contamination in viticulture. Unlike traditional methods that rely on laboratory-based testing or require expert intervention, our approach uses low-cost environmental sensors, such as those measuring temperature and humidity, to monitor conditions related to OTA growth. The data collected by these sensors are processed through a neural network model deployed on a microcontroller designed for real-time operation. This model classifies the likelihood and concentration of OTA contamination at various stages of grape harvesting, enabling proactive intervention. Our work introduces a novel end-to-end system that can classify OTA contamination levels based on real-time environmental data, offering a scalable, cost-effective, and autonomous solution for grape farmers. This system eliminates the need for specialized equipment and domain expertise, making it accessible on-site. The dataset used to train the model is derived from an analytical feasibility study on grape samples, providing a solid analytical foundation for understanding OTA growth dynamics. By focusing on real-time data processing, our system can predict OTA contamination risks as they emerge, offering a preventive tool for ensuring food safety. Furthermore, the implementation of this system on a resource-constrained microcontroller demonstrates its feasibility for large-scale deployment in field conditions. This work sets a new precedent in the application of AI and IoT in viticulture, providing an affordable and efficient method to combat OTA contamination and improve food safety in grape and wine production. Our paper is structured into three main sections. In Section 2 the state-of-the-art related to electronic-based solutions for mycotoxins identification is presented. In Section 3, starting from the analytical feasibility study on grape samples, the novel method is introduced, encompassing discussions on hardware and software components, as well as the implementation of the predictive model within the hardware framework. Moreover, in Section 4, we describe our experimentation process and present the results obtained. Final remarks are presented in Section 5.

## 2. State of the Art

Mycotoxins, particularly OTA, pose significant health risks, necessitating comprehensive monitoring strategies to prevent their occurrence in food products. The presence of mycotoxins in foodstuffs is heavily influenced by environmental conditions such as humidity and temperature, which are critical for their growth. Traditional detection methods, although highly accurate, involve chemical analyses that are generally complex and costly for continuous monitoring. For instance, High-Performance Liquid Chromatography (HPLC) with fluorescence detection [22,23] offers precise quantification of mycotoxins like OTA but requires skilled personnel and specialized lab equipment, limiting its accessibility for on-site testing and rapid analysis in field settings. Liquid Chromatography-Mass Spectrometry (LC-MS) [24], particularly with electrospray ionization, provides high sensitivity and accuracy, enabling the detection of OTA even at very low concentrations. However, this technique demands advanced instrumentation and controlled laboratory conditions, making it challenging to implement for routine on-site testing. Thin-Layer Chromatography (TLC) [25] is a simpler and more cost-effective method for the preliminary screening of OTA, suitable for use in coffee-producing countries. While effective for detecting OTA at levels like 5 mg/kg, it does not offer the precision required for more detailed quantitative analysis compared to advanced methods like HPLC. These conventional techniques offer valuable accuracy but are often limited by high costs and the need for specialized settings, making them impractical for continuous, field-based monitoring. In response to these limitations, non-chemical approaches, particularly those integrating IoT and AI, are emerging as practical alternatives for real-time, on-site monitoring. The authors in [26] used AI and IoT technologies through the ISCA-MobileNetV3 model combined with an artificial olfactory sensor (AOS) to achieve over 98.5% accuracy in detecting mycotoxin-contaminated maize while reducing olfactory arrays from 16 to 6 with minimal accuracy loss. This system demonstrated rapid online evaluations (<1 s) and offers a low-cost, high-precision solution for real-time maize quality detection. Another paper [27] addresses the growing concern over food quality and safety in the context of socio-economic development, highlighting the limitations of traditional food analysis methods. It proposes a comprehensive approach integrating AI and biosensors for more effective food quality evaluation. The paper discusses the potential prospects, challenges, and future research directions in this field. Noor et al. [28] developed a portable electrochemical biosensor integrated with an IoT cloud system for in situ detection of multi-mycotoxins, including aflatoxin B1 (AFB1) and OTA, both of which are harmful types of mycotoxins. The device successfully developed standard curves for both toxins, demonstrating its capability for multi-mycotoxin detection, although field testing focused on AFB1 in grain corn. While the device effectively detected AFB1, its reliance on a stable internet connection for IoT functionalities may limit its use in remote areas. Additionally, Akhtar et al. [29] developed an IoT system that utilizes environmental sensors and an Arduino UNO microcontroller to detect conditions conducive to fungal growth, which can lead to mycotoxin production. This system continuously monitors environmental parameters, such as precipitation and CO_2_ levels, identifying any notable patterns or variations that might be connected to fungal activity. With real-time monitoring and analysis, the technology can quickly identify the presence of fungi and ensure the security of stored food products. Similarly, the authors in the paper [30] propose a compact convolutional transformer (CCT) model to classify wheat contamination by mycotoxins, achieving an accuracy of 83.33%. The model effectively detects contaminated, incipient, and healthy wheat based on CO_2_ respiration rates and mold formation. It highlights the need for further improvement in distinguishing between healthy and incipient classes for timely spoilage detection. As part of our research, we conducted a comparison between the Random Forest (RF) model and fully Connected Neural Networks (CNNs). While the training and validation results of RF were comparable to those of CNNs, the RF model, like other relevant algorithms, such as Support Vector Machines (SVM), k-Nearest Neighbors (k-NN), and Gradient Boosting Machines (GBM), generally exhibits a larger size after conversion [31]. This makes such models less suitable for deployment on resource-limited devices like the M5Stick C Plus (M5Stack Technology Co., Ltd., Shenzhen, China) (M5C+). Additionally, CNNs offer significant advantages in terms of adaptability, particularly with techniques like transfer learning, aligning with our goal of developing a generalized and scalable solution. Our study builds upon these developments, specifically targeting the critical factors of temperature, humidity, and harvest time duration, which are the main determinants of OTA growth in grapes. We employ low-cost IoT sensors to monitor these parameters continuously, providing a direct, feasible solution for detecting and managing OTA risks in the field. By focusing on the environmental measurements that directly affect OTA growth, our research fills a crucial gap in current mycotoxin detection strategies, offering a cost-effective, scalable solution adaptable to varying field conditions. This approach not only complements existing technologies but also extends their capabilities to create a more integrated and effective monitoring system. However, there are limitations that need to be considered, such as the use of the M5C+ device in our system requires recharging before each harvesting cycle, as its current battery life can support up to two harvest cycles at most. Additionally, while our focus was on developing a proof of concept, the data flow was not optimized, suggesting that there is plenty of room for optimization regarding protocols and data transmission for more robust, efficient, or secure data transmission that will be the focus in future implementations.

## 3. Methods

**Figure 1:**
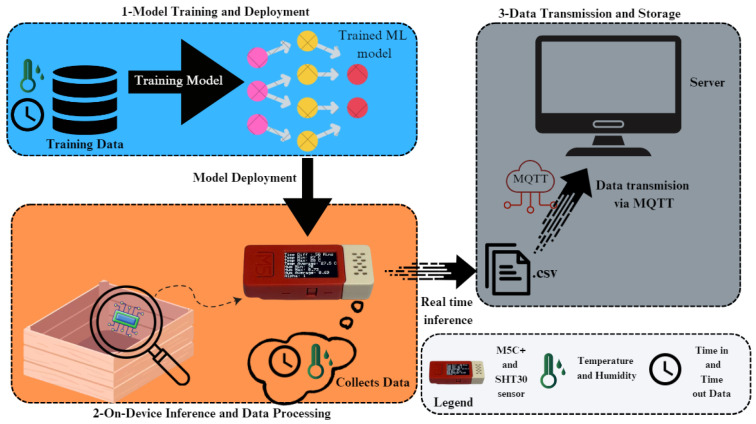
Overview of the proposed system’s architecture, detailing three main sections: (1) Model training and deployment; (2) On-device inference and data processing; (3) Data transmission and storage.

**Figure 2:**
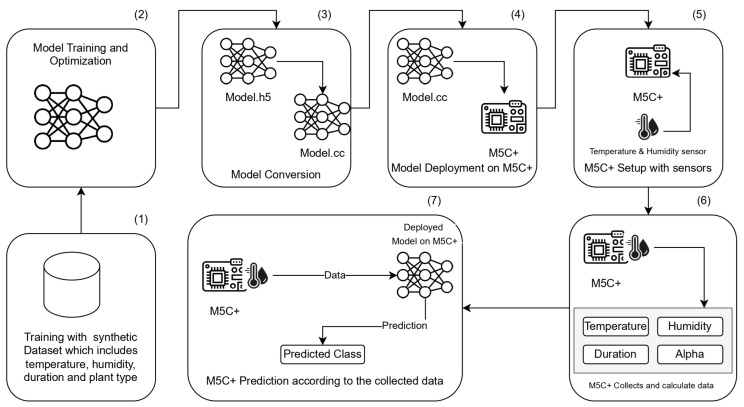
AI model flowchart showing the workflow from training to deployment for OTA prediction. The model is trained and optimized in (2) on a synthetic dataset in (1), converted from .h5 to .cc in (3), and deployed on an M5C+ device in (4). The M5C+ is setup with sensors in (5) for real-time data collection in (6) and OTA concentration prediction in (7).

In this section, we will detail the proposed system architecture designed to estimate the concentration level of OTA in harvested white grapes. In addition, the description of implementation and the deployment of the models on a M5C+ device is provided. We explain how the model was conceived, trained, and the expected working mode, detail the device used, and outline the process of embedding the model into the device. The organization of this section is as follows: The system shown in Figure 1 starts by using synthetic data generated to simulate real-world environmental conditions, such as temperature, humidity, and time. These synthetic data are used to train an ML model offline. The training and deployment process shown in Figure 2 involves feeding the data into the ML model to enable it to learn patterns and relationships within the dataset, preparing it for inference tasks. Once the model is trained, it is optimized and deployed onto the M5C+ microcontroller. Then, the trained ML model is integrated into the M5C+, which serves as an edge device for real-time data processing, which is placed in or attached to a box. The M5C+ collects live environmental data using its attached sensors, simulating the conditions represented by the synthetic data. It processes these data locally on the device, performing on-device inference to classify the collected data into the correct class based on the model knowledge. Finally, the classified data are transmitted to a server using the MQTT protocol for lightweight and efficient communication. The transmitted data are saved in a (.csv) file format on the server, where they can be further analyzed or visualized. An analytical feasibility study to explain the developed modelization that was conducted on white grape samples is provided in Section 3.1. It is followed by a detailed description in Section 3.2 of the used hardware devices. Following this, the software implementation is introduced in Section 3.3, providing a detailed description of the dataset creation from the analytical feasibility study on grape samples, moving to model design and model optimization, and concluding with a description of the implementation of the proposed model in hardware in Section 3.4.

### 3.1. Evaluation Function of Contamination by OTA in Grapes

An analytical feasibility study, based on a mycological survey, to explain the developed predictive model was conducted on white grape samples. The extraction of OTA was carried out according to the VICAM method developed by Rosa et al. in [32]. To 5 mL of the sample (juice or pulp), 10 mL of a solution containing H3PO4–NaCl was added and mixed for 1 min. The resulting sample was then supplemented with 5 mL of chloroform, and the mixture was centrifuged at 2500× *g* for 15 min. The organic phase at the bottom was transferred to a flask. The extraction was repeated a second time, and the two organic phases were combined and dried. The dried extract was reconstituted in a PBS solution containing 15% (*v*/*v*) methanol and transferred to the immunoaffinity column (Ochratest, VICAM). The column was pre-washed with 20 mL of PBS. The sample was loaded into the immunoaffinity column, and the operation was repeated three times with 5 mL of PBS-methanol solution each time. The column was then washed with water and dried. OTA was eluted with 1.5 mL of methanol (HPLC grade). The collected eluate was dried under a nitrogen stream and reconstituted with 50 μL of the mobile phase.

#### 3.1.1. HPLC-Determination

The analyzes were carried out with a Shimadzu Nexera UHPLC-RF system (Shimadzu, Kyoto, Japan), composed of a controller (CBM-20A), a degasser (DGU-20A5R), dual-plunger parallel-flow pumps (LC-30AD), an autosampler (SIL-30AC), a column oven (CTO-20AC), and a fluorimetric detector (RF-20A). The column used was a Phenomenex Luna 5 μm C18 (150 × 4.60 mm). The mobile phase employed was a mixture of acetonitrile:water:acetic acid in a ratio of 99:99:2, with a flow rate of 0.8 mL/min. The excitation wavelength was set at 333 nm, and the emission wavelength at 477 nm. OTA was identified using the external standard method. LC data processing was performed with LC solution software (Version 5.71, Shimadzu).

#### 3.1.2. Optimization of Method

The calibration curve was constructed using the least squares method by obtaining the equations of the regression lines. Mandel’s test confirmed the linearity of calibration curve to be in the considered range (0.25–10 g/L). The limit of quantification (LoQ) and limit of detection (LoD) were calculated by multiplying the standard deviation (SD) of the lowest level of the calibration curve ten and three times, respectively, and dividing the result for the slope of the calibration curve. The values were 0.08 μ/L for LOD and 0.12 μ/L for LOQ. Instrumental recovery of 94% was also determined.

#### 3.1.3. Results

**Figure 3:**
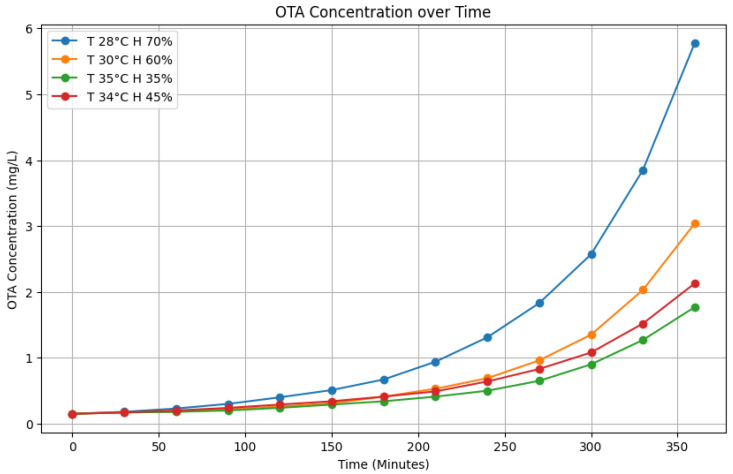
Evaluation function over time for OTA.

In this part, which represents a feasibility study, a predictive model was built. Through the building of the model, a method for correlating the OTA amount to different fungal development variables was developed. In Figure 3, the graphical trend of OTA production during the harvest time at different humidity and temperature conditions is reported. Considering the average trend of temperature and relative humidity during the period and within the grape harvesting area, the following pairs of variables were selected and evaluated: 30 °C temperature and 60% of humidity, 34 °C and 45%, 35 °C and 35%, 28 °C and 70%. These variables were selected to evaluate the increase in the OTA amount during the time (6 h). As can be seen in the Figure 3, the OTA amount increased quickly at 28 °C with the humidity of 70% corresponding to night hours (from 8 pm to 7 am). Instead, harvesting when the temperature is up to 35 °C but humidity is at 35% [21] would lead to an optimal product harvest with lower OTA content.

### 3.2. Hardware

**Figure 4:**
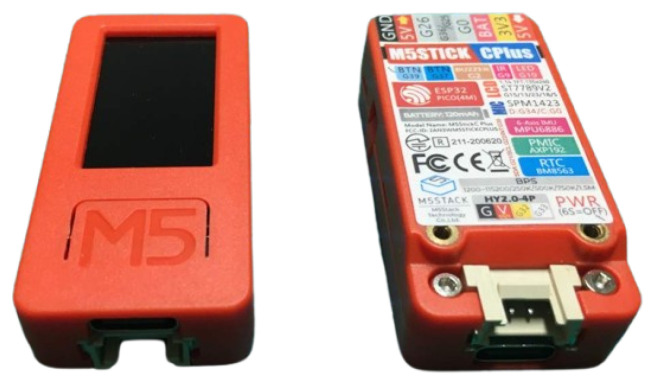
M5stick C plus [36].

The M5C+ device shown in Figure 4 was selected for the IoT implementation for its effectiveness, portability, and versatility. We opted for the M5C+ due to its robust capabilities, perfectly aligned with the requirements of the project. Its integrated architecture, encompassing an ESP32 microcontroller, built-in display, and an array of sensors, streamlines development while offering ample room for expansion [33]. Moreover, the M5C+ proves to be an optimal choice for rapid prototyping and deployment, owing to its intuitive user interface and extensive community support [34]. Leveraging tools such as VS Code, Python, and Arduino, along with the M5C+ Core development environment, and equipped with built-in Wi-Fi support and compatibility with emerging technologies like LoRaWAN, our system seamlessly transmits data over MQTT, facilitating real-time communication and remote monitoring [35]. Furthermore, we used the low-cost sensors provided by M5C+ that are easy to use and able to ensure precise data acquisition. In particular, the built-in 6-Axis IMU acts as a trigger to start data acquisition and the ENV-III Hat contains the digital humidity and temperature sensor SHT30 (Sensirion AG, Staefa ZH, Switzerland) from Sensirion. In the intended application, when the grapes are harvested, the M5C+ is firmly placed in or attached to a box containing the grapes. Subsequent to a box tilt, precisely when the box is flipped upside down for more than five seconds according to the accelerometer measurements, the data acquisition process starts. On-line measurement and saving on the on-device memory of the temperature and humidity values through the SHT30 sensors are then performed every 60 s. This time duration provides a good balance between the need for acquiring enough measurements to model the process and the requirements of reducing the dissipated energy for every acquisition, and the amount of used memory. Additionally, the M5C+’s generous memory and clock speed ensure that our classification model operates with proper efficiency and accuracy, facilitating effective real-time data measurement, classification, and transmission.

#### Details of the M5C+

Table 1 lists some of the key characteristics of the M5C+:

### 3.3. Software

A proper dataset that contains the underlying connections among the measurable harvesting conditions and the OTA contamination probability obtained from the analysis presented in Section 3.1 is requested to train an ML model. Embedding an ML model on a microcontroller offers significant advantages over deterministic functions, particularly for complex and dynamic problems [10]. ML models excel in handling non-linear relationships, adapt to new data, and efficiently process noisy sensor inputs, making them ideal for resource-constrained environments where adaptability and efficiency are crucial.

#### 3.3.1. Dataset Description

The dataset was designed to synthesize time-interval-based weather data for applications requiring temporal and environmental correlations. It simulates real-world temperature and humidity measurements. The considered temperature range varies from 28 °C to 35 °C, and the humidity ranges from 35% to 70%, reflecting daily weather data of the village of Bianco in Reggio Calabria gathered from [37]. The days considered [21] were used for white grapes harvesting. The temperature and humidity range also coincides with the limit of the environmental conditions considered in the quantitative measurements performed in Section 3. By mapping weather data to randomly generated time periods with realistic durations (30–360 min), the dataset mirrors operational scenarios, such as agricultural planning.

The synthetic dataset consists of the following features:

- **TimeIn**: Start time of the interval.
- **TimeOut**: End time of the interval.
- **Tavg**: Average temperature during the interval.
- **Havg**: Average humidity during the interval.
- **Alpha**: A derived parameter based on environmental and temporal factors.
- **Label**: Classification or evaluation value assigned to the interval.

This dataset ensures realistic variability by simulating weather data and aligning it with typical operational constraints.

#### 3.3.2. Dataset Labeling Function

The labeling of dataset samples in this study is based on a mathematical function that estimates OTA concentration as a function of time, temperature, and humidity. The labeling process involves two main steps: (1) Calculating the OTA concentration using a weighted model and (2) assigning labels to the dataset based on predefined concentration ranges.

##### Concentration Estimation

The OTA concentration is calculated using an exponential growth model and a weighted interpolation between different environmental conditions, extracted from the OTA concentrations (in Figure 3) at different temperatures and humidity. The base model for OTA concentration under a specific condition is given as:

(1) $$C_{\mathrm{condition}(t)}=a\cdot e^{b\cdot t}+c$$

where:

- *t*: Time in minutes
- a,b,c: Curve-fitting parameters specific to the environmental condition
- *T*: Temperature
- *H*: Humidity.

To generalize this model across multiple environmental conditions, weights are introduced based on the proximity of the sample’s temperature *T* and humidity *H* to the reference temperature $T_{\mathrm{ref}}$ and humidity $H_{\mathrm{ref}}$ of each condition. The weight for a condition is calculated as:

(2) $$w_{\mathrm{condition}}\left(T,H\right)=\frac{\exp\left(-\frac{{\left(T-T_{\mathrm{ref}}\right)}^{2}}{10}-\frac{{\left(H-H_{\mathrm{ref}}\right)}^{2}}{100}\right)}{\sum_{\mathrm{condition}}\exp\left(-\frac{{\left(T-T_{\mathrm{ref}}\right)}^{2}}{10}-\frac{{\left(H-H_{\mathrm{ref}}\right)}^{2}}{100}\right)}$$

The overall OTA concentration is then computed as a weighted sum:

(3) $$C\left(t,T,H\right)=\sum_{\mathrm{condition}}w_{\mathrm{condition}}\left(T,H\right)\cdot C_{\mathrm{condition}}\left(t\right)$$

##### Environmental Conditions

The dataset considers the following environmental conditions, each characterized by a reference temperature and humidity:

- **T28_H70: **$T_{\mathrm{ref}}=28\;{}^{\circ}C,H_{\mathrm{ref}}=70\%$
- **T30_H60: **$T_{\mathrm{ref}}=30\;{}^{\circ}C,H_{\mathrm{ref}}=60\%$
- **T35_H35: **$T_{\mathrm{ref}}=35\;{}^{\circ}C,H_{\mathrm{ref}}=35\%$
- **T34_H45: **$T_{\mathrm{ref}}=34\;{}^{\circ}C,H_{\mathrm{ref}}=45\%$

##### Binary Classification

In binary classification, samples are labeled based on whether the OTA concentration is within acceptable production limits. Specifically, concentrations below or equal to 2 (C≤2) are considered acceptable and labeled as class 0, while concentrations above 2 (C>2) are labeled as class 1. The binary labeling function is defined as:

(4) $${\mathrm{Label}}_{\mathrm{binary}}\left(C\right)=\left\{\begin{array}{cc} 0, & \mathrm{if}\;C\leq 2\\ 1, & \mathrm{if}\;C>2 \end{array}\right.$$

This binary labeling approach ensures that the dataset aligns with real-world production criteria, distinguishing between acceptable and unacceptable OTA levels in wine and grape juice production, according to Commission Regulation (EC) No. 1881/2006 [38].

##### Multi-Class Classification

For multi-class classification, samples are divided into five classes based on predefined OTA concentration ranges. This allows for finer-grained labeling of the dataset. The multi-class labeling function is defined as:

(5) $${\mathrm{Label}}_{\mathrm{multi}\text{-}\mathrm{class}}\left(C\right)=\left\{\begin{array}{cc} 0, & \mathrm{if}\;0\leq C\leq 1\\ 1, & \mathrm{if}\;1<C\leq 2\\ 2, & \mathrm{if}\;2<C\leq 3\\ 3, & \mathrm{if}\;3<C\leq 4\\ 4, & \mathrm{if}\;C>4 \end{array}\right.$$

The multi-class labeling provides additional granularity for applications where the precise level of OTA concentration is relevant.

##### Implementation

The dataset labeling function integrates the concentration estimation and label assignment steps. The model calculates the OTA concentration for each sample and assigns a corresponding label, enabling the generation of a robust labeled dataset suitable for training and evaluating machine learning models.

#### 3.3.3. Dataset Structure

- Anomaly Detection Dataset (ADD)
  Firstly, by using the function described in Section 3.3.2, we generated a dataset with two classes: class ‘0’ encompasses below the threshold OTA concentrations, which are safe for the production of wine and grapes juice [38], while class ‘1’ comprises the above threshold concentrations, which are not safe for production, as shown in Table 2.
- Probability Classification Dataset (PCD)
  Secondly, using the function described in Section 3.3.2, a novel dataset with five classes was created. The concentrations of each class are described in Table 3.

#### 3.3.4. Dataset Generation Process

The dataset generation process begins by defining *N*, the total number of samples to generate. For each sample, random time intervals, *TimeIn* ($T_{\mathrm{in}}$) and *TimeOut* ($T_{\mathrm{out}}$), are selected within the range of 3:00 AM to 8:00 PM. The duration of these intervals is computed in minutes as:

(6) $$Time_{\mathrm{Difference}}=T_{\mathrm{out}}-T_{\mathrm{in}}$$

During each time interval, temperature and humidity data are processed to compute the following features:

- $Time_{\mathrm{Difference}}$
- $T_{\mathrm{Average}}$
- $H_{\mathrm{Average}}$

Using these features, a concentration value is calculated using an evaluation function Section 3.3.2:

(7) $$C(Time_{\mathrm{Difference}},T_{\mathrm{average}},H_{\mathrm{average}}).$$

Based on the computed concentration, a label is assigned to each sample following the dataset structure. Finally, each sample, containing $Time_{\mathrm{Difference}}$, $T_{\mathrm{Average}}$, $H_{\mathrm{Average}}$, and α, is added to the synthesized dataset. The algorithm used to initialize and generate the dataset is shown in Algorithm 1.
**Algorithm 1 **Dataset generation algorithm**Input:** None // No input data**Output:** generatedDataset // Synthesized Dataset1:**Initialization:** Set *N* // Number of samples to generate2:Set α=1 // The algorithm can be personalized based on different plant conditions3:**for **i=1 to *N* **do**4:    Pick Random TimeIn;5:    Pick Random TimeOut;6:    Calculate TimeDifference: i[TimeDifference]=(TimeOut−TimeIn) (in minutes);7:    Compute i[TemperatureAvg] for the duration from TimeIn to TimeOut;8:    Compute i[HumidityAvg] for the duration from TimeIn to TimeOut;9:    Calculate i[Concentration] using the function:10:           i[Concentration]=C(i[TimeDifference],i[TemperatureAvg],i[HumidityAvg],α);11:   Assign i[label] based on i[Concentration] using the dataset structure;12:   Add *i* (containing TimeDifference, TemperatureAvg, HumidityAvg, α, and Lable) to generatedDataset;13:**end for**

#### 3.3.5. AI Model Design

**Figure 5:**
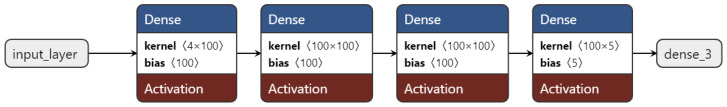
PCD model architecture.

Two fully connected neural networks with identical architectures were employed, one for PCD shown in Figure 5, and the other for ADD. We began with the architecture described in [21], and through iterative development, adapted it to improve the performance. This process led to our current architecture, which is detailed as follows: Each network is composed of an input layer followed by three hidden layers, each with 100 neurons. These layers are fully connected. The input layer directly receives the features: $Time_{\mathrm{Difference}},T_{\mathrm{average}},H_{\mathrm{average}},$ and α, constrained to one plant type for simplicity. The ADD model’s output layer uses a sigmoid activation function, suitable for binary classification by providing an output that represents a probability between 0 and 1, with a total of 20,902 parameters. In contrast, the PCD model employs a softmax activation function in its output layer, which converts network output into a probability distribution across 5 classes. In the hidden layers, the ReLu activation function was selected to facilitate learning of non-linear relationships without the complications of vanishing gradients. The PCD model has a total of 21,205 parameters. Both models utilize sparse categorical cross-entropy for the loss function and the Adam optimizer, reflecting the best practices in current neural network training. For the training of these models, a number of epochs equal to 250 was appropriate for both ADD and PCD, as shown in Table 4. This parameter was determined after observing consistent improvements in training performance up to this point. As shown in Table 4 and Table 5, specifically, the ADD model reached its optimal training accuracy of 99.29% with loss of 0.219 at epoch 215 and attained a validation accuracy of 99.71% with a validation loss of 0.012, while the PCD model achieved a peak training accuracy of 97.28% with a loss of 0.707 at epoch 229, and a validation accuracy of 98.55% with a validation loss of 0.060. The validation set, being equal to 20% of the size of the training set, presents a smaller and potentially less complex data distribution, making it easier for the model to achieve higher accuracy and lower loss on the validation set compared to the training set. To significantly enhance the validity of the training results, we performed training using k-fold cross-validation with a rate of 5, as detailed in Table 6. This iterative development from the initial architecture in [21] was crucial in tailoring a solution that efficiently addresses both classification and detection challenges in our dataset. For the given models, we calculate the Floating Point Operations (FLOPs) for each layer [39]. The FLOPs for a fully connected layer are given by:

$$FLOPs_{\mathrm{layer}}=2\times \left(\mathrm{Number}\;\mathrm{of}\;\mathrm{inputs}\times \mathrm{Number}\;\mathrm{of}\;\mathrm{outputs}\right)$$

ADD Model Calculation:

- Input Layer: 4 inputs (since we have 4 features).
- Hidden Layers: 3 hidden layers, each with 100 neurons.
- Output Layer: 1 output (binary classification).

**First Layer (Input → Hidden Layer 1):**

FLOPs=2×4×100=800

**Second Layer (Hidden Layer 1 → Hidden Layer 2):**

FLOPs=2×100×100=20,000

**Third Layer (Hidden Layer 2 → Hidden Layer 3):**

FLOPs=2×100×100=20,000

**Fourth Layer (Hidden Layer 3 → Output Layer):**

FLOPs=2×100×1=200

**Total FLOPs for ADD Model:**

$$FLOPs_{\mathrm{ADD}}=800+20,000+20,000+200=41,000\;\mathrm{FLOPs}$$

PCD Model Calculation:

- Input Layer: 4 inputs.
- Hidden Layers: 3 hidden layers, each with 100 neurons.
- Output Layer: 5 outputs (5-class classification).

**First Layer (Input → Hidden Layer 1):**

FLOPs=2×4×100=800

**Second Layer (Hidden Layer 1 → Hidden Layer 2):**

FLOPs=2×100×100=20,000

**Third Layer (Hidden Layer 2 → Hidden Layer 3):**

FLOPs=2×100×100=20,000

**Fourth Layer (Hidden Layer 3 → Output Layer):**

FLOPs=2×100×5=1000

**Total FLOPs for PCD Model**:

$$FLOPs_{\mathrm{PCD}}=800+20,000+20,000+1000=41,800\;\mathrm{FLOPs}$$

#### 3.3.6. Model Optimization

To optimize the model, we focused on the training process and the organization of the data. The model was trained for 250 epochs to achieve high accuracy. A comparative analysis showed that part of the features used in the first study [21] were not providing any additional values; for this reason, we decided to apply a feature selection and to reduce up to four input features, which were $Time_{\mathrm{Difference}},T_{\mathrm{average}},H_{\mathrm{average}}$, and the α parameter. From the beginning of the development activity, we were aware of the eventual deployment on the M5C+ device. This foresight allowed us to tailor the architecture to fit precisely within the device’s constraints, such as its limited memory and computational capabilities. We carefully designed the structure of the layers to ensure the model’s efficient operation within an edge computing environment [10]. By optimizing the model to fit the requirements of the M5C+, we facilitated the conversion process to C without compromising the accuracy of the model’s outputs while maintaining essential features and representations. These preemptive optimizations make this implementation uniquely suited for resource-constrained environments, offering an advantage by harmoniously balancing model size with predictive performance. This approach preserves the accuracy and integrity of our model’s predictions while ensuring smooth integration into C-based frameworks.

### 3.4. End-to-End ML Model in Hardware

The implementation of our classification/detection models in M5C+ required a number of crucial actions to guarantee smooth operation and end-to-end integration within the M5C+ device. A summary of the procedure is as follows:

- ML model conversion to C: the trained models were converted into a compatible format for the programming environment of the M5C+. To enable effective execution on microcontrollers, this usually entails translating the model into C code. In our application, we exploited TensorFlow Lite for Microcontrollers [40] to integrate the ML-model-to-C conversion into our M5C+ device. With the help of this library, microcontrollers with limited memory and processing power may load and run ML models [41].
- Library setup: TensorFlow Lite libraries, to enable model inference on the M5C+.
- ML model loading: upload of the ML model to the device’s flash memory.
- Firmware adaptation to integrate (i) the starting trigger from the built-in IMU, (ii) the saving of humidity and temperature samples every 10 s, (iii) the on-board inference, and (iv) the output and MQTT transmission of the result.

Moreover, the measurements and the model classification results are shown on the M5C+’s built-in display.

Upon booting the code on the M5C+, it occupies 110,740 out of 327,680 bytes in the RAM, which is 33.80% of the total available RAM. Additionally, regarding the FLASH memory, the code occupies 511,153 out of 1,310,720 bytes, representing 39.00% of the total available flash memory. Furthermore, the inference and detection time from the M5C+ was measured using an oscilloscope, ranging from 11 millisecond (ms) to 12 ms, drawing only approximately 2.75 millijoules (mJ) of energy.

## 4. Experimentation and Results

In this section, along with a comparison with previous findings, we describe our experimental design and results. We highlight the advantages and disadvantages of our methodology by contrasting our results with those of other studies on OTA contamination in grape cultivation.

### 4.1. Compliance Verification Using Grape Harvest Dataset from Reggio Calabria

**Figure 6:**
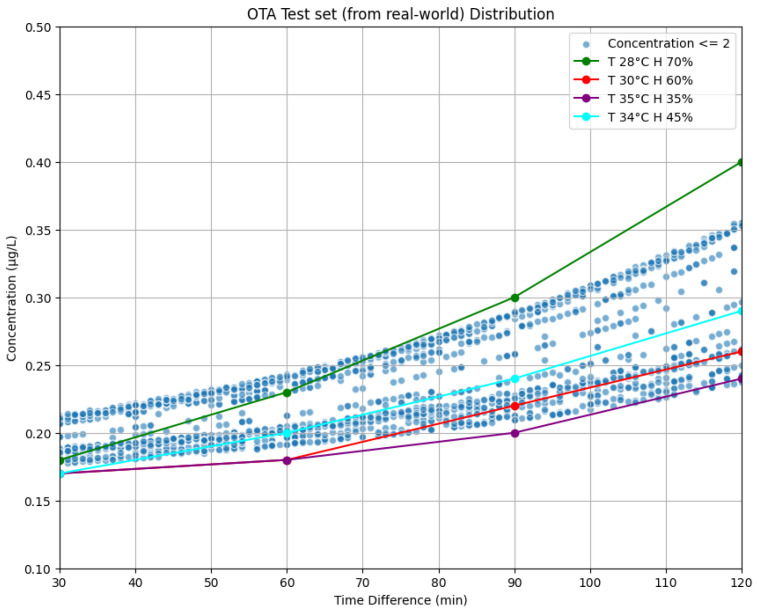
A test set (from real-world) distribution.

In our study, we further evaluated the practical applicability of the OTA concentration prediction function described in Section 3.3.2 by calculating the theoretical concentration for actual grape harvesting operations for which we knew the referred temperature and humidity distributions. The field operations refer to real-world farming activities operated in the village of Bianco in Reggio Calabria [37]. The related weather data were specifically gathered on the specific days when local farmers harvested white grapes [21], providing a precise context for assessing the influence of environmental factors on OTA concentrations. This dataset, used for illustrative purposes, was processed to demonstrate how OTA concentration levels vary over time under actual field conditions, while consistently respecting the defined concentration boundaries, as suggested from the practical experience of the farmers. Figure 6 displays the distribution of the samples, showcasing the results of the concentration prediction function described in Section 3.3.2 when applied to this comprehensive real-world test set. This visualization not only highlights the potential utility of our function as an analytical tool in the agrifood sector but also confirms that the concentration levels consistently remain at or below the regulatory limit of 2 mg/L, a critical threshold for the safe production of grape juice and wine. This real-world test set, representing days when grapes are harvested and coupled with corresponding weather data, was used exclusively to validate the effectiveness of the concentration function described in Section 3.3.2, underscoring its accuracy and reliability in practical scenarios. For comprehensive testing of the models, we generated two additional datasets that included samples from all classes in both cases of ADD and PCD.

### 4.2. Test Set Generation

For the ADD model, we generated a test set where each class contained 2000 samples, resulting in a total of 4000 samples. Similarly, for the PCD model, with 5 classes, the test set contained 2000 samples per class, resulting in a total of 10,000 samples.

### 4.3. Test Set Distribution

**Figure 7:**
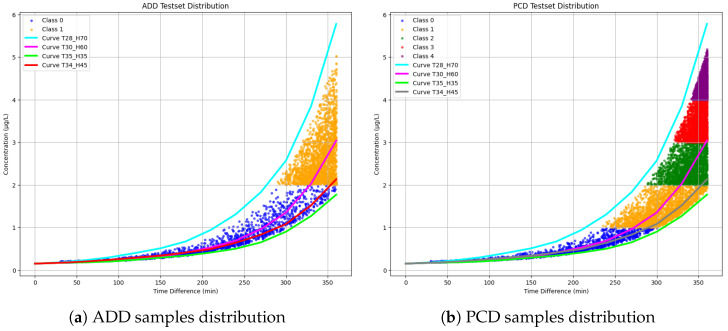
Test set samples distribution: ADD and PCD.

Before testing our models on the two different test sets, we analyzed the distribution of the test sets elements by plotting them on the graph presented earlier in Figure 3. This visualization aimed to provide a clear understanding of how the test samples are distributed relative to the function. Figure 7a illustrates the distribution of the ADD test set samples, labeled with their respective concentrations, overlaid on the function plot. Similarly, Figure 7b presents the distribution of the PCD test set samples, also labeled with their concentrations, on the corresponding function plot.

### 4.4. Model Testing

**Figure 8:**
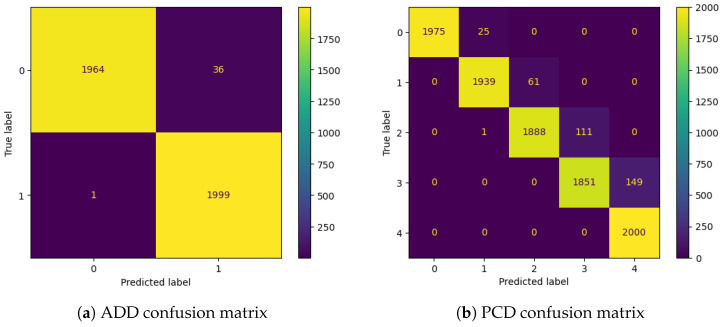
Confusion matrixes.

By using the generated test sets, the ADD model demonstrated remarkable performance in binary classification, accurately classifying 3963 out of 4000 samples, as reported in the confusion matrix shown in Figure 8a. This corresponds to a test accuracy of 99.07%. Similarly, the PCD model, evaluated on its testset, correctly classified 9653 out of 10,000 samples, as shown in Figure 8b, achieving a test accuracy of 96.53%.

### 4.5. Results

**Figure 9:**
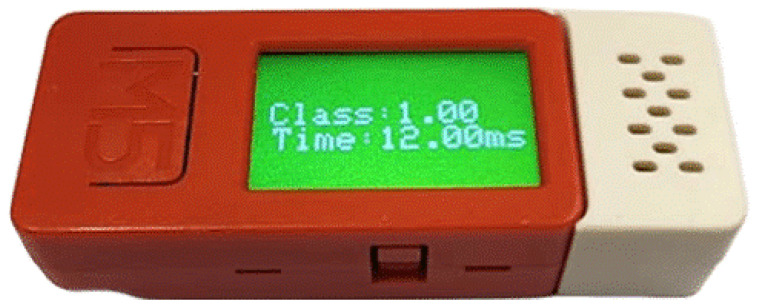
Classification results using M5C+. The screen shows the predicted class and the inference time in milliseconds.

Based on environmental conditions, our proposed approach is able to provide a real-time classification of OTA contamination into two classes for the AAD model and five classes for the PCD model. Our approach accurately classifies samples by taking into account time and variable environmental parameters as temperature and humidity. This strategy makes it possible to implement focused intervention plans and improves our capacity to reduce the hazards brought on by mycotoxin contamination in agrifood products. The results of classification are shown in the built-in display of the device, to easily prevent the supply of contaminated grapes, as shown in the Figure 9, where the device inferences are classified according to the data collected.

After the deployment of our classification models on the M5C+ device, we conducted two tests to evaluate the battery consumption. Both tests were performed with the LCD display turned off to minimize power consumption, and data transmission was performed via MQTT after every inference. The first test involved the device performing inferences every 30 min, which corresponds with the minimum time required for harvesting operations. Over the course of 6 h, the battery level decreased from 96.87% to 55%. In the second experiment, the device was set to perform inferences every 60 min. This less frequent inference schedule aimed to observe the effects on battery longevity when reducing operational demand. Over the same 6 h period, the battery level decreased from 97.23% to 63.32%. These results demonstrate a significant reduction in battery life even with less frequent inferences, highlighting the energy demands of real-time data processing and transmission in field conditions.

**Figure 10:**
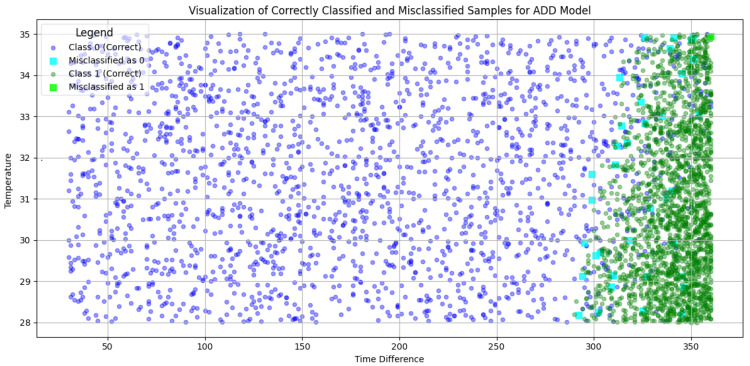
Classification and measurement results using M5C+ for ADD.

**Figure 11:**
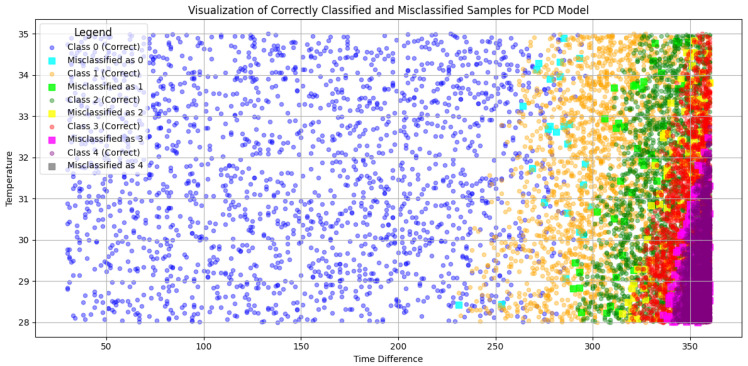
Classification and measurement results using M5C+ for PCD.

Building on this, the results of our classification approach are detailed in Figure 10 and Figure 11, showcasing the model’s performance in identifying mismatches in both binary and multi-class setups. Figure 10 focuses on the binary classification results using the ADD model. It visualizes the separation between two contamination levels based on time and concentration, highlighting regions where the model misclassifies samples. The accompanying confusion matrix quantifies these mismatches, emphasizing the model’s strong performance, with minimal misclassifications. These results demonstrate the ADD model’s effectiveness in discerning contamination levels while also identifying specific areas for further improvement. Figure 11 presents the multi-class classification results using the PCD model, where OTA contamination is categorized into five distinct classes based on time and concentration. The visual representation shows the distribution of correctly classified and misclassified samples across the feature space, while the confusion matrix provides a detailed breakdown of these mismatches. Some mismatches are concentrated in certain classes, indicating areas where the model encounters difficulty in differentiating adjacent contamination levels. This is particularly evident in elements located on the borders between classes, where classification becomes challenging due to their ambiguous positioning. However, not all borders elements are mismatched, some generated elements in the test set are inherently difficult to classify due to the nature of the dataset. Both figures highlight the utility of the proposed approach in providing detailed insights into the classification process, ensuring the supply of uncontaminated grapes and enhancing food safety in the agrifood supply chain.

**Figure 12:**
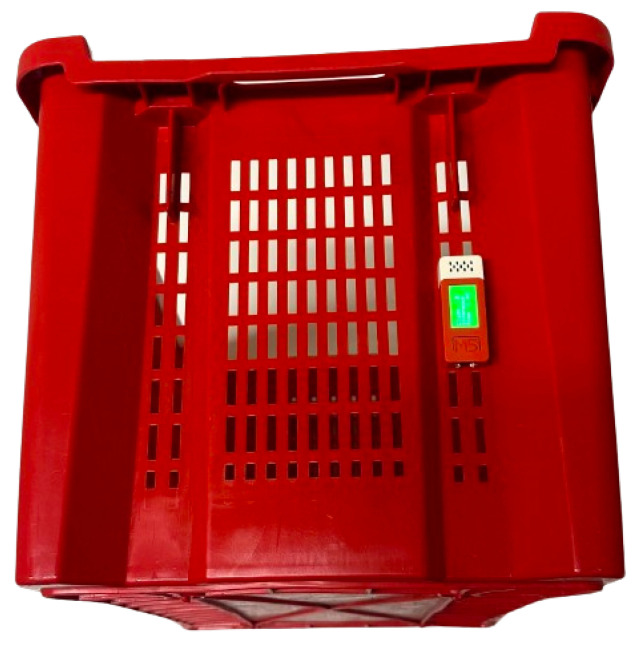
Commercial harvesting box with integration of the M5C+.

The proposed approach shows several results that indicate potentiality for its on-field implementation:

- In the end-to-end pipeline of the application building, the first step conceived a thorough chemical study that is reflected in a modelization of the contamination phenomena. This result was used to carefully label the datasets used to train the embedded ML model, which guarantees accurate model building and validation right away.
- A compact wearable system integrates M5C+ hardware with environmental sensors, designed to measure and quantify various environmental conditions. This system features a display on the M5C+ that shows the collected measurements. Additionally, it includes a classification model specifically designed to evaluate these situations and categorize samples appropriately. The model operates with an inference time ranging between 11 and 12 ms and consumes only a few μW.
- The ML model on the M5C+ device can be updated remotely, which facilitates easy and effective maintenance and upgrades and avoids model drift.
- AI is integrated into this system to guarantee consistent performance and continuous improvement, enabling it to handle massive amounts of data and adapt to changing environments, and requiring a limited bandwidth only to update the inference results. ML models are used to enhance coordination and functioning so that every component of the IoT ecosystem communicates in a cohesive manner.
- The position of the M5C+ (shown in the Figure 12) within the system is adaptable and not strictly fixed, allowing it to be adjusted according to specific applications or use cases. This flexibility ensures that the M5C+ location can be optimized based on environmental factors, ease of access, or data transmission needs, enhancing its effectiveness across various deployment scenarios.
- The system is conceived to be easily deployed, guaranteeing user-friendliness. In Figure 12, an example of integration of the device on a commercial box is presented.
- The analysis also underscores the capability of our OTA concentration prediction function to enhance food safety and quality within agrifood practices. Displayed through the application of data from a grape harvesting day in Bianco in Reggio Calabria, the function effectively integrates into existing workflows. By providing producers with crucial information on OTA concentrations, our approach supports informed decision-making and proactive management of contamination risks. This integration ensures adherence to safety standards, demonstrating the value of our function in maintaining compliance with regulatory limits for OTA concentrations, crucial for the safe production of juice and wine.

## 5. Conclusions

This study demonstrates a novel application of AI and IoT technologies for the early assessment of OTA contamination risks in grape cultivation, offering a practical and low-cost solution to enhance food safety and quality. The NN model, deployed on a resource-constrained M5C+, leverages temperature, humidity measurements, and the time of harvesting as primary inputs. We used this limited set of two sensors to maintain affordability and accessibility, and our results indicate that this approach can achieve performance similar to traditional OTA risk assessment techniques when considering the limitations of those techniques, as discussed in Section 2. While this two-sensor setup effectively monitors conditions favorable to OTA growth, the model is adaptable and can be enhanced by incorporating additional sensors, such as those for light exposure, carbon dioxide, oxygen levels, water activity, and environmental stressors. These added inputs would provide a more comprehensive understanding of OTA risk factors, allowing for improved accuracy and adaptability across diverse environmental scenarios. This study not only demonstrates the effectiveness of our approach in comparison to traditional methods but also highlights its efficiency. By utilizing a minimal set of resources, materials, and expert interventions, we offer a swift and practical solution for on-field applications, with inference times ranging from a negligible 11 ms to 12 ms. This rapid assessment capability significantly enhances decision-making processes in the field. This study remains open to other developments that aim to mimic different conditions and achieve similar results with traditional techniques while preserving the low-cost concept. By providing a solution that improves monitoring in the agrifood sector, we contribute to consumer safety and create an adaptable, scalable tool. Moreover, this approach can be expanded for broader applications in the agrifood sector, allowing for the detection and management of various contaminants or conditions affecting quality and safety across different crops and supply chains. Future work will focus on extending the functional boundaries of the system by broadening its range to cover a wider spectrum of environmental conditions, such as temperatures beyond the current 28 °C to 35 °C range and humidity levels beyond 35% to 70%. Additionally, chemical analyses will be employed to refine and adapt the system for enhanced functionality, ensuring its applicability to a more diverse set of environmental scenarios and plant types. Future developments will also address the current limitation of battery consumption by the M5C+ device, aiming to enhance its operational efficiency and reduce the need for frequent recharging, ensuring uninterrupted field operations, by exploiting sustainable and renewable solar powered sources.

## Figures and Tables

**Table 1:** Characteristics of M5C+.

Feature	Description
Microcontroller	ESP32-PICO-D4 (240 MHz dual core, 600 DMIPS)
Flash Memory	16 MB
RAM	8 MB (520 KB SRAM)
Display	0.96-inch TFT color display (80 × 160 resolution)
Battery	95 mAh Li-Po battery
Connectivity	Wi-Fi (802.11 b/g/n), Bluetooth 4.2 BLE
Expansion	Grove compatible connector
Sensors	Accelerometer (MPU6886), IR transmitter
Input/Output	Built-in buttons (2), microphone, speaker
Interfaces	USB-C for charging and programming
Dimensions	48 mm × 24 mm × 13.5 mm
Weight	Approximately 14 g

**Table 2:** ADD Structure.

Concentration of OTA	Concentration (mg/L)	Class	Production Safety
**Below threshold**	0.00 ≤ Concentration ≤ 2.00	0	Safe
**Above threshold**	Concentration > 2.00	1	Unsafe

**Table 3:** PCD Structure.

Concentration of OTA	Concentration (mg/L)	Class	Production Safety
**Approaching zero**	0.00 ≤ Concentration ≤ 1.00	0	Safe
**Low**	1.00 > Concentration ≤ 2.00	1	
**Moderate**	2.00 > Concentration ≤ 3.00	2	Unsafe
**High**	3.00 > Concentration ≤ 4.00	3	
**Critical**	Concentration > 4.00	4	

**Table 4:** Training results table displaying performance metrics obtained on the train set.

Model	Train Accuracy	Loss	Number of Samples	Number of Samples per Class
**ADD**	99.29%	0.219	25,000	12,500
**PCD**	97.28%	0.707	25,000	5000

**Table 5:** Validation results table displaying performance metrics obtained on the validation set.

Model	Validation Accuracy	Validation Loss	Number of Samples	Number of Samples per Class
**ADD**	99.71%	0.012	5000	2500
**PCD**	98.55%	0.060	6250	1250

**Table 6:** Model performance using k-fold cross-validation.

K-Fold Rate	Accuracy (%)		Loss	
	ADD	PCD	ADD	PCD
5	98.93 ± 0.32	96.59 ± 1.04	0.033	0.081

## Data Availability

Data are contained within the article.
